# A Case Report of Pulmonary Arterial Hypertension Associated With Hereditary Hemorrhagic Telangiectasia Successfully Treated With Riociguat

**DOI:** 10.7759/cureus.94152

**Published:** 2025-10-08

**Authors:** Toshiki Sakuma, Toshihiko Sugiura, Keiko Yamamoto, Akira Naito, Ayumi Sekine, Ayako Shigeta, Takuji Suzuki

**Affiliations:** 1 Department of Respiratory Medicine, Graduate School of Medicine, Chiba University, Chiba, JPN

**Keywords:** arteriovenous malformations, hereditary hemorrhagic telangiectasia, pulmonary arterial hypertension, pulmonary hypertension, pulmonary vasodilator

## Abstract

Hereditary hemorrhagic telangiectasia (HHT) is a rare vascular disorder that is occasionally complicated by pulmonary hypertension (PH). In most cases where PH is associated with HHT, it is typically of a post-capillary type, while pre-capillary PH is relatively rare. Herein, we report a rare case of pre-capillary PH in a 44-year-old woman. She presented with recurrent epistaxis, mucocutaneous telangiectasias, a family history of hemorrhagic events, and hepatic arteriovenous malformations. She met the four Curaçao criteria and was diagnosed with HHT. Right heart catheterization demonstrated severe pulmonary arterial hypertension in the absence of portal hypertension. Given the risks of bleeding, management of both HHT and PH presents notable challenges. In this case, treatment with riociguat improved pulmonary vascular resistance (PVR) without exacerbating epistaxis or causing other adverse effects. These findings suggest that riociguat may represent a promising therapeutic option for HHT-associated pre-capillary PH.

## Introduction

Hereditary hemorrhagic telangiectasia (HHT) is a rare autosomal dominant disorder characterized by systemic vascular malformations, with an estimated prevalence of one to two per 10,000 individuals [1]. The disease results in mucocutaneous telangiectasias and visceral arteriovenous malformations (AVMs), leading to a broad spectrum of clinical manifestations ranging from recurrent epistaxis to critical solid organ bleeds. Pulmonary hypertension (PH), which was first reported in a patient with HHT in 1969 [2], is a chronic progressive condition that is increasingly being recognized as a clinical complication of HHT, with a reported prevalence of approximately 15% [3]. Typically, PH in HHT is post-capillary, developing as a consequence of high-output cardiac states caused by significant shunting through large visceral AVMs, particularly in the liver. In contrast, pre-capillary PH resembling pulmonary arterial hypertension (PAH) represents a distinct and less common form, thought to arise from intrinsic pulmonary vasculopathy [4]. Therefore, distinguishing PAH from post-capillary PH and establishing optimal management strategies remains a critical clinical challenge.

## Case presentation

A 44-year-old woman was referred to our hospital, a PH and liver-lung transplant facility, for further evaluation of suspected PH. Abnormalities on electrocardiography (ECG) and chest radiography during a health checkup prompted a visit to a local clinic, where transthoracic echocardiography revealed findings suggestive of PH. She experienced daily episodes of epistaxis, which also occurred in her father and older sister. Her father had a history of cerebral hemorrhage. She had no remarkable past medical history.

**Figure 1:**
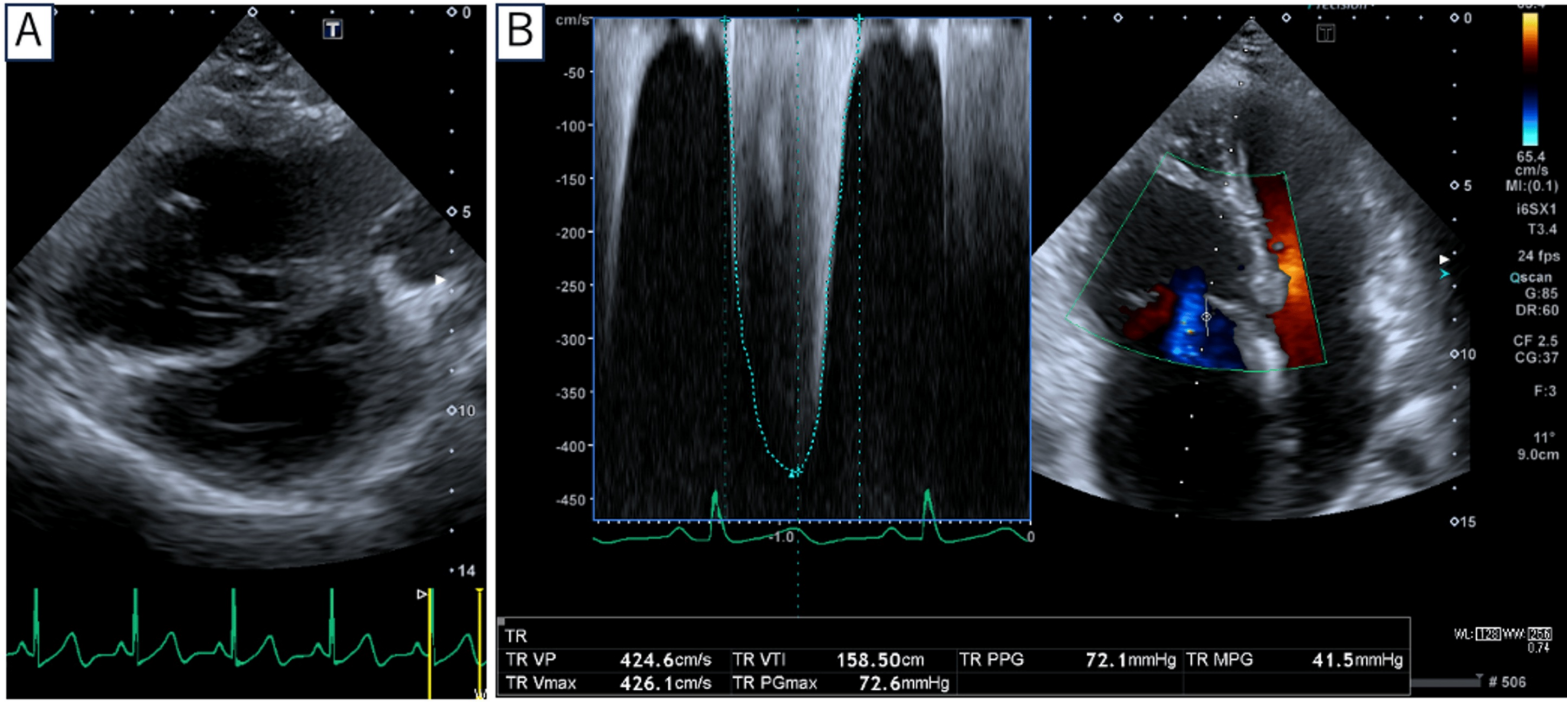
Transthoracic echocardiography. (A) Short-axis view demonstrating a D-shaped left ventricle. (B) Apical four-chamber view showing tricuspid regurgitation pressure gradient of 72 mmHg.

**Figure 2:**
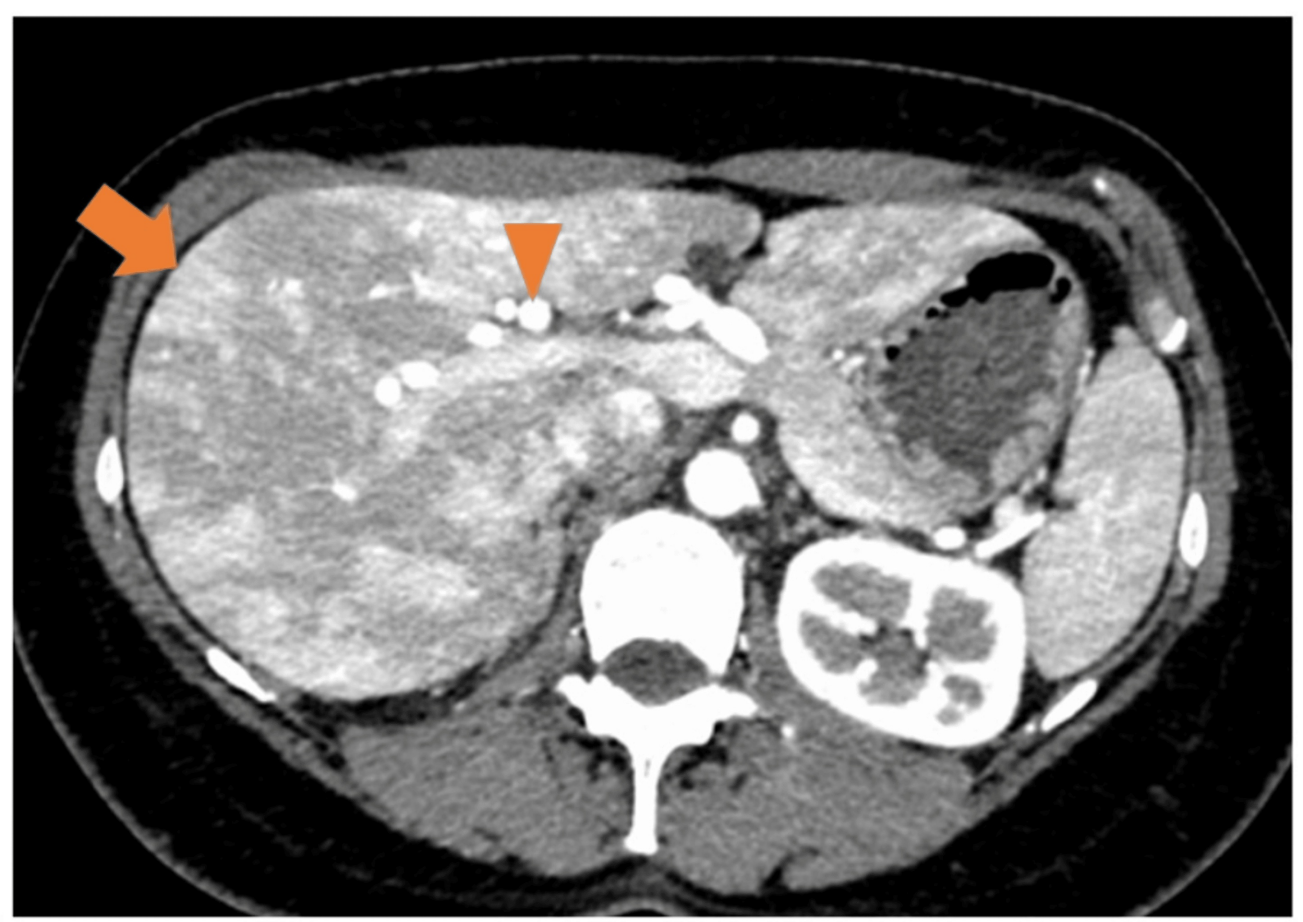
Early phase of dynamic liver computed tomography (CT) shows a wedge-shaped enhancement (arrow) and hepatic artery dilatation (triangle).

**Figure 3:**
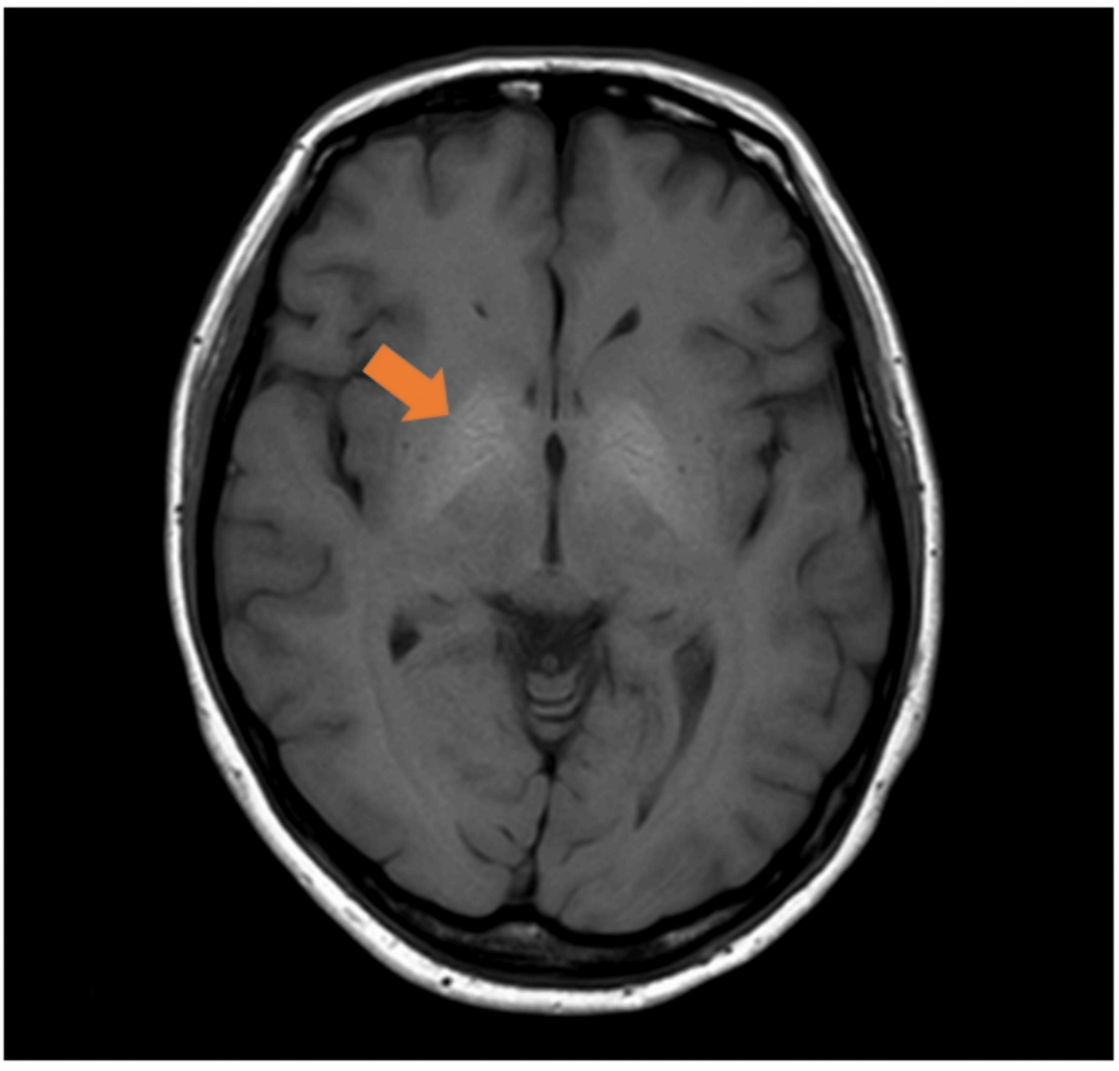
Brain magnetic resonance imaging (MRI) shows T1 hyperintense enhancement in the globus pallidus (triangle).

Physical examination revealed telangiectasias on the skin of the fingers and chest. Oral examination revealed no telangiectasias. Laboratory and functional assessment at baseline revealed: hemoglobin 13.3 g/dl, hematocrit 40.6%, mean corpuscular volume (MCV) 91.4 fL, platelet count 305 × 103/µL, serum iron 105 µg/dL, ferritin 30.8 ng/mL, transferrin saturation 32.3%, aspartate aminotransferase (AST) 23 IU/L, alanine aminotransferase (ALT) 19 IU/L, serum creatinine 0.61 mg/dL, and B-type natriuretic peptide (BNP) 183.9 pg/mL. The six-minute walk distance was 411 m. The patient was WHO Functional Class I at presentation. The ECG showed inverted T waves in leads II, III, aVf, and V1-V5, suggesting right ventricular strain. Chest radiography revealed enlargement of the pulmonary arteries with a cardiothoracic ratio of 55%. In addition, pulmonary function testing revealed a reduced diffusion capacity for carbon monoxide at 11.74 mL/minute/mmHg (55% predicted), and transthoracic echocardiography revealed right ventricular enlargement and a tricuspid regurgitation pressure gradient of 72 mmHg (Figure 1). Echocardiography revealed no valvular heart disease, left ventricular dysfunction, or intracardiac shunt. A ventilation-perfusion scan revealed no ventilation-perfusion mismatch. Contrast-enhanced computed tomography of the chest revealed no pulmonary AVMs; however, hepatic artery enlargement and an arterioportal shunt were noted (Figure 2). Moreover, brain magnetic resonance imaging (MRI) showed T1 hyperintense enhancement in the globus pallidus, consistent with manganese deposition secondary to a hepatic portosystemic shunt (Figure 3). Right heart catheterization revealed a pulmonary artery pressure (PAP) of 93/29 mmHg (mean 58 mmHg), a pulmonary artery wedge pressure (PAWP) of 9 mmHg, a cardiac index (CI) of 3.14 L/minute/m², and a pulmonary vascular resistance (PVR) of 8.91 Wood units. Liver venous pressure measurements obtained during catheterization revealed a wedged hepatic venous pressure of 11 mmHg, free hepatic venous pressure of 10 mmHg, and hepatic venous pressure gradient of 1 mmHg, thereby indicating an absence of portal hypertension.

On the basis of the presence of spontaneous recurrent epistaxis, mucocutaneous telangiectasias, a first-degree relative with similar symptoms, and hepatic AVMs, the patient met the four Curaçao criteria and was diagnosed with HHT. Moreover, on the basis of the hemodynamic findings, the patient was diagnosed with HHT complicated by PAH. Risk stratification using the REVEAL 2.0 score resulted in a total of six points, corresponding to the low risk category [5].

Treatment with riociguat was initiated, with the dosage being gradually titrated to a maximum dose of 7.5 mg/day. During the course of treatment, there was no evidence to indicate an exacerbation of epistaxis or progression of anemia, and follow-up right heart catheterization revealed an improvement in PVR (Table 1); however, PVR remained at an elevated level, prompting the addition of a second agent. Although the patient was referred to and attended our genetic counseling department, she declined to undergo genetic testing.

**Table 1:** Patient’s hemodynamic parameters. Pre-treatment: diagnosis in room air. Post-treatment: four months after the initiation of riocigut in room air. CI: cardiac index; mPAP: mean pulmonary arterial pressure; PAWP: pulmonary artery wedge pressure; PVR: pulmonary vascular resistance; RAP: right arterial pressure; WU: Wood unit

Parameters	Pre-treatment	Post-treatment
RAP (mmHg)	8	9
mPAP (mmHg)	58	48
PAWP (mmHg)	9	11
CI (L/minute/m^2^)	3.14	2.89
PVR (WU)	8.91	7.37
PaO_2_ (mmHg)	54	69
PvO_2_ (mmHg)	45	43
PaCO_2_ (mmHg)	36	40

## Discussion

PH associated with HHT can be classified into two major types: post-capillary and pre-capillary. In the majority of cases in which PH is associated with HHT, it is of the post-capillary type, typically resulting from high-output states attributable to hepatic AVMs or anemia. In contrast, pre-capillary PH resembling PAH is relatively rare, occurring in less than 1% of cases [4]. Pre-capillary PH is characterized by obliterative plexiform vasculopathy of the small pulmonary arteries and is often ascribed to an underlying genetic abnormality, such as mutations in the ACVRL1 gene. The pathology of pre-capillary PH in HHT is similar to that of PAH [6]. In addition, this spectrum also includes pulmonary veno-occlusive disease (PVOD) and pulmonary capillary hemangiomatosis (PCH), in which vasodilator therapy may precipitate pulmonary edema [7,8]. Chronic thromboembolic pulmonary hypertension (CTEPH) is another important differential diagnosis. Our case illustrates the diagnostic challenge of distinguishing pre-capillary PH from post-capillary PH in HHT. Although the patient had hepatic AVMs that could have contributed to a high-output state, right heart catheterization demonstrated pre-capillary hemodynamics (mPAP > 20 mmHg, PVR > 2 Wood units, and PAWP ≤ 15 mmHg), confirming the diagnosis of PAH. PVOD/PCH and CTEPH were effectively ruled out by chest CT findings and ventilation-perfusion scintigraphy, respectively. This underscores the importance of invasive hemodynamic testing, as the presence of AVMs may be misleading. Importantly, embolization of hepatic AVMs is generally not recommended, even in patients with a high-output state, because of the high risk of severe hepatic complications [9].

At present, there are no established treatments for PAH in patients with HHT. Some case studies have reported improvements in response to certain pharmacological interventions, including the use of bosentan, sildenafil, selexipag, epoprostenol, treprostinil, and iloprost [10-13]. However, given potential bleeding complications, the management of PAH during HHT can be extremely challenging, and pulmonary vasodilators may also increase the risk of bleeding. In particular, prostacyclin pathway agents inhibit platelet aggregation, and phosphodiesterase inhibitors may worsen epistaxis [14,15]. Additionally, endothelin receptor antagonists carry the risk of causing anemia, irrespective of bleeding [16]. Accordingly, given these concerns, in the present case, we selected riociguat, based on the consideration that its stepwise dose escalation enabled a vigilant assessment of epistaxis. Notably, the patient exhibited a clear hemodynamic response to this treatment, with a reduction in PVR and no deterioration of epistaxis or anemia during treatment. These findings suggest that riociguat could be a promising therapeutic option.

The patient presented with severe PH at baseline, and the 2022 ECS/ERS guidelines typically recommend dual or triple combination therapy [15]. However, starting with multicombination therapy from the beginning may pose difficulties due to complications of HHT. Lyle et al. reported that among 10 patients with HHT who received PAH-specific therapy (eight with PAH and two with post-capillary PH), five were forced to discontinue treatment due to bleeding events [17]. Therefore, the decision to pursue sequential therapy for our case was made. While our patient responded to monotherapy, her PVR remained elevated, promoting the addition of a second agent. For patients with inadequate response to multi-drug therapy, bilateral lung transplantation may be considered.

## Conclusions

Accurate hemodynamic assessment by right heart catheterization is essential for the diagnosis of pre-capillary PH and to differentiate it from post-capillary PH, particularly in the presence of visceral AVMs. Our findings suggest that riociguat may potentially be an effective and well-tolerated option for the treatment of PAH in patients with HHT. Further research on similar cases is required to validate the efficacy and safety of this drug.
